# Previous Foreign Body Ingestion in the Appendix Causing Acute Appendicitis: A Case Report

**DOI:** 10.7759/cureus.34948

**Published:** 2023-02-13

**Authors:** Mohammad Hamadneh, Maysaa Al-Khalaileh, Aseed Alayed, Farah R Barhoush, Sereena Hijazin, Jorgeat Haddad, Mohammad Abu-Jeyyab

**Affiliations:** 1 Pediatrics and Neonatology, South Shouna Hospital, Amman, JOR; 2 General Surgery, Al-Basheer Hospital, Amman, JOR; 3 School of Medicine, Mutah University, Al-Karak, JOR; 4 General Member, Mutah Research and Audit Society, Amman, JOR; 5 Emergency Medicine, King Abdullah University Hospital (KAUH), Irbid, JOR; 6 Coordinator, Mutah Research and Audit Society, Amman, JOR

**Keywords:** needle, perforation, asymptomatic, foreign body, appendicitis

## Abstract

Acute appendicitis due to a foreign body is a very rare condition with an incidence of 0.0005% of all appendicitis cases and among all age groups. It is one of the atypical cases of appendicitis, and it is a rare condition commonly asymptomatic; there is a period of time between ingestion of the body and appendicitis.

A 14-year-old female patient presented to the emergency room with right lower quadrant colicky pain. Moreover, it was progressive with six hours duration, preceded by loss of appetite. It was associated with nausea, non-projectile vomiting, and diarrhea. By taking the past medical history, the patient had a history of multiple times of foreign ingestions when she was younger. On examination, the patient appeared ill, and was vitally stable. On palpation, the patient had a right lower quadrant tenderness. The patient had positive pointing, rebound, Rovsing, and psoas signs. Full labs were done. Abdominal x-ray revealed a radiopaque metallic body in the right lower quadrant. By ultrasound, there was a minimal free fluid collection in the pelvis. Intraoperatively, the appendix looked hyperemic. Appendectomy was performed, and a needle was extracted from the appendix. Furthermore, the histopathology revealed an early inflamed appendix.

Foreign body-causing appendicitis is a rare condition. We need to investigate suspected cases carefully because the presentation is atypical, and sometimes the patients are asymptomatic.

## Introduction

The appendix is a vestigial hollow organ located at the tip of the cecum and is most commonly present in the right lower quadrant of the abdomen [1]. Acute appendicitis is an inflammation of the appendix. It occurs in various age groups, mostly between 10 and 20 years old, while foreign-body-induced appendicitis is known to occur more often within the pediatric population. Furthermore, appendicitis is more common in males, as the lifetime risk of acute appendicitis reaches 8.6% in males and 6.9% in females [2]. A typical case of appendicitis would present with an initial periumbilical pain that localizes to the right lower quadrant. In addition, the pain is usually accompanied by fever, nausea, vomiting, and anorexia [3]. However, foreign-body-induced appendicitis has a wider range of presentations, from completely asymptomatic, to abdominal pain and other nonspecific symptoms [4]. Appendicitis is most likely caused by a blockage of the appendix's lumen, mainly by a fecalith. However, it rarely happens by an ingested foreign body, as demonstrated in our case. It is scarce for ingested foreign bodies to reside in the appendix, for most of them would pass through the gastrointestinal tract asymptomatically and without complications [4]. It has been noted that foreign-body ingestion is quite a common problem within the pediatric population and that foreign-body-induced appendicitis presents in only 0.005% of cases [5].

Moreover, the incidence varies with the appendix's position and the appendiceal orifice's size. As for the complications, they would also vary depending on the ingested body’s nature. Some blunt objects are more susceptible to remaining dormant for years, in contrast to sharp objects, which cause higher rates of perforation and peritonitis [6]. In this case, we discuss how a previously known asymptomatic ingestion of a foreign body precipitated appendicitis two years after ingestion.

## Case presentation

A 14-year-old female patient presented to our emergency department with a complaint of a right lower quadrant progressive abdominal pain over six hours.

The patient described a six-hour history of periumbilical pain that shifted to the right iliac fossa, which began suddenly after some hours of loss of appetite. The pain was colicky and constant, with no irradiation. In addition to that, she experienced an associated feeling of hotness and nausea with vomiting twice that day. The non-projectile vomitus was approximately in a volume of a cup of tea related to the food she had eaten before with no blood. The patient was having associated diarrhea (loose stools many times). She mentioned no changes in the frequency of urination, dysuria, or change in urine color. The patient's first menstrual period was when she was 12 years old. The period was regular with two days of bleeding every 28 days with mild bleeding, three pads/day, and mild dysmenorrhea.

The patient reported a free medical surgical and family history of any medical illnesses. However, the patient reported a history of non-symptomatic needle ingestion two years ago.

By doing the relative physical examination, the patient looked ill; the abdomen was moving with respiration. It was symmetrical on both sides, non-distended (normal flanks), and had a normal central inverted umbilicus. However, by palpation, she had a tender guarding right lower abdomen. Additionally, she had positive pointing, rebound, Rovsing, and psoas signs.

Furthermore, the gynecological consult revealed that there was no apparent gynecological condition that could be correlated with the patient's complaint.

The patient’s vital signs at the admission were temperature: 38 C, pulse: regular 110 beats per minute, respiratory rate: 19 breaths per minute, blood pressure: 103/75 mmHg, and oxygen saturation: 95%. The pain score was 6/10.

Hematology (complete blood count) and biochemistry (kidney and liver function) tests were within the reference ranges. A urine analysis was unremarkable, with no positive findings.

**Figure 1:**
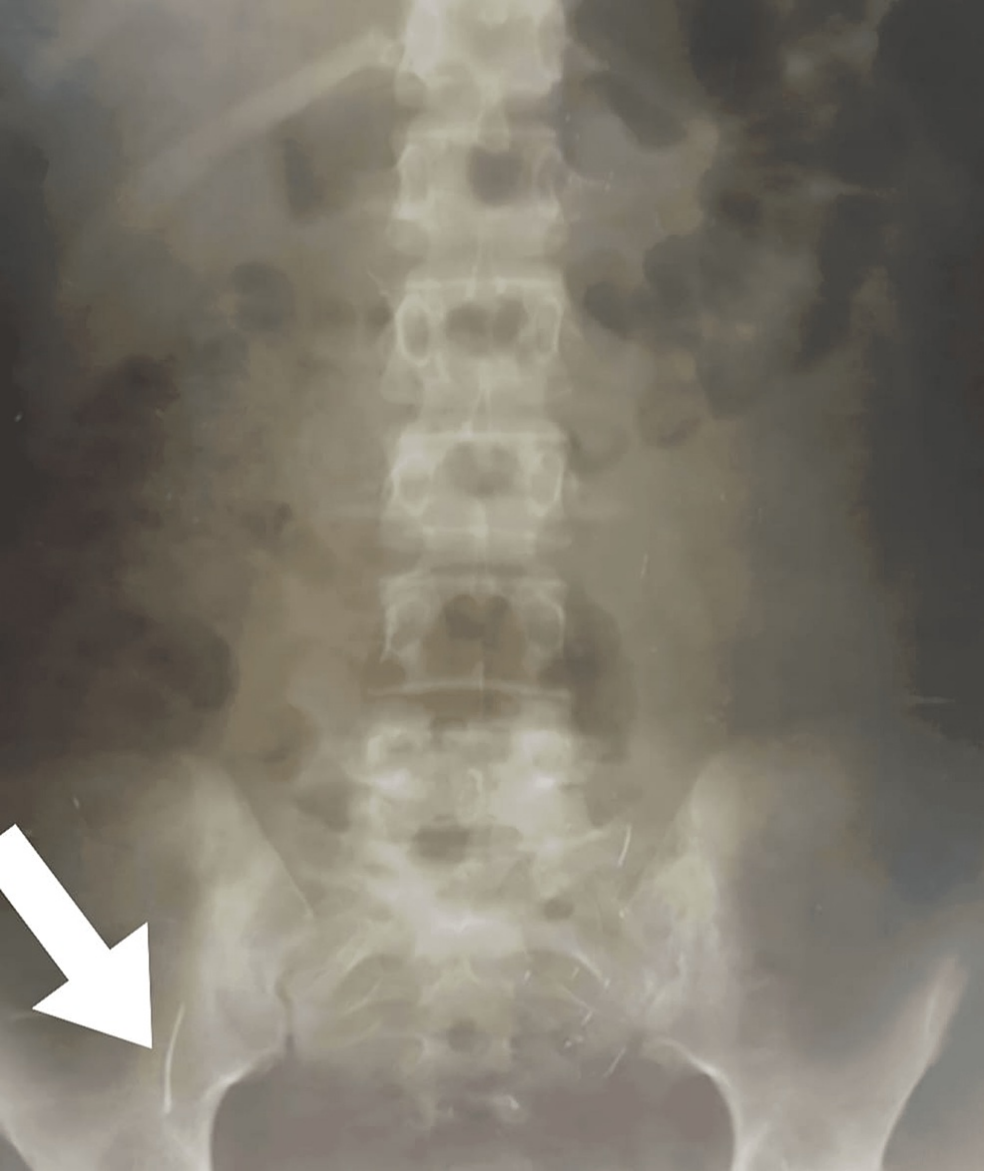
Erect abdomen x-ray. The arrow shows the needle opacity in the appendix.

Ultrasonography was obtained, and it reported nonspecific findings, including a minimal free fluid collection in the pelvis, despite negative ultrasonographic signs of appendicitis because the appendix could not be visualized. Nevertheless, the plain abdominal x-ray showed a radiopaque metallic foreign body in the right lower quadrant (Figure 1). 

**Figure 2:**
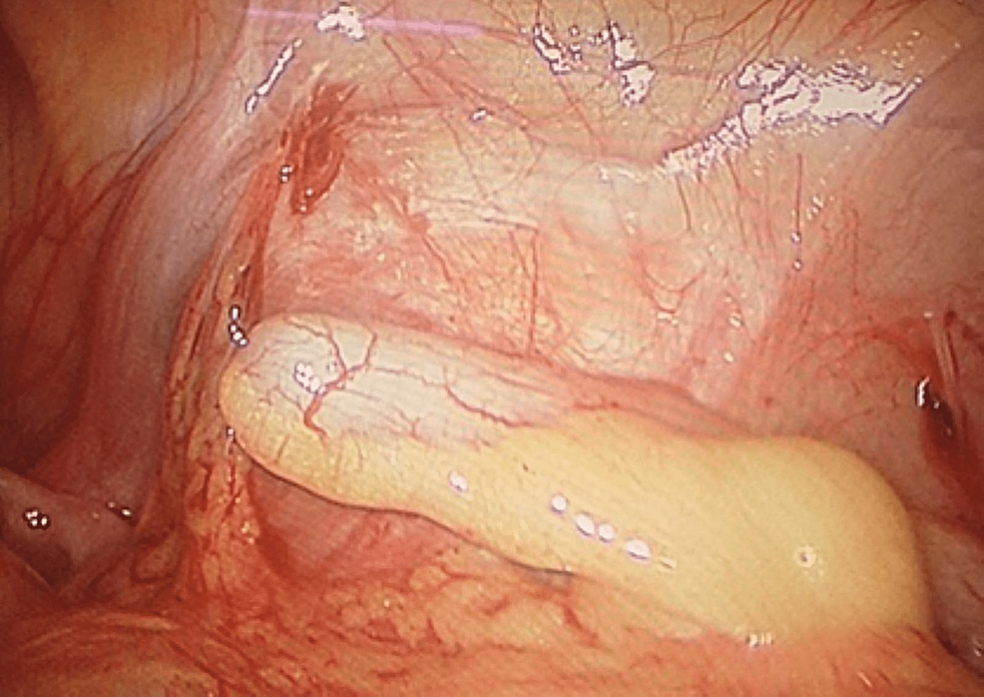
Intraoperative laparoscopic view of the appendix.

**Figure 3:**
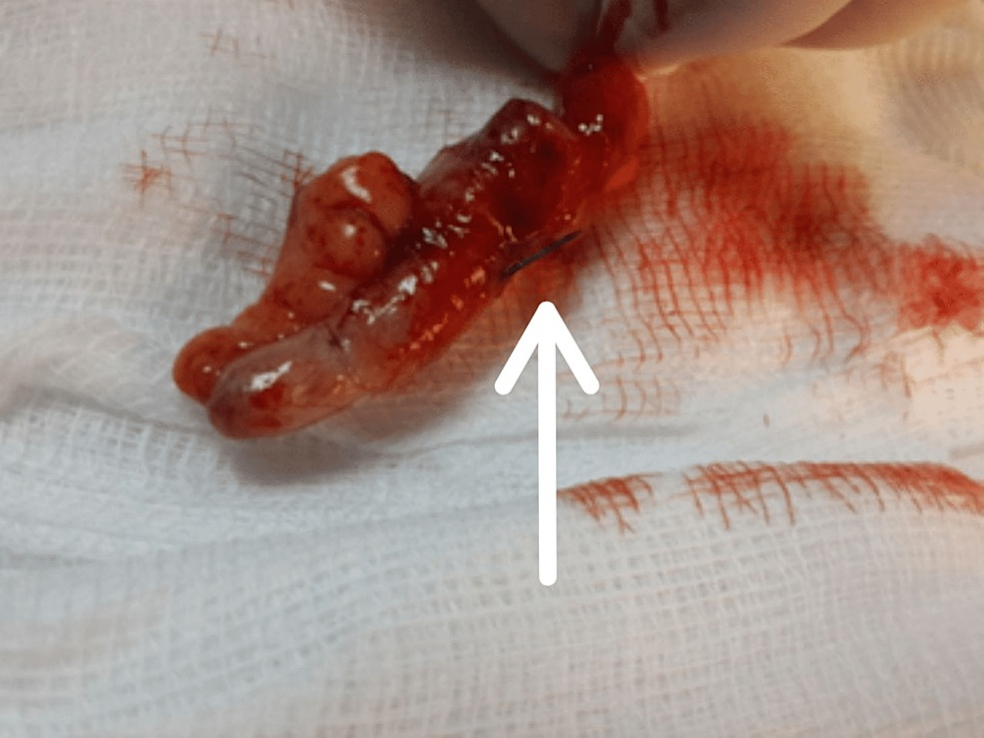
Gross view of the appendix post appendectomy with the needle penetrating the appendix.

Consequently, laparoscopic exploration and subsequent appendectomy for the hyperemic appendix penetrated with a sharp foreign body were performed (Figure 2). Grossly, a needle was extracted from the appendix (Figure 3). Post-operative recovery passed smoothly with a satisfactory outcome. The histopathological examination reported signs of an early inflamed appendix with no signs of malignancy at all.

## Discussion

With a global incidence of 100/100000 across all age categories, appendicitis is one of the most prevalent surgical emergencies [7]. There are many different causes of appendicitis or inflammation of the appendix, including bacterial growth within the appendix and obstruction of the appendix lumen by calcified fecaliths or bezoars, tumors, lymphadenitis, and foreign bodies. This blockage leads to filling the appendix with mucus, leading to increased pressure within the appendicular lumen. It will decrease blood flow to the tissue and cause bacterial growth inside the appendix, causing inflammation [8]. However, foreign bodies are a rare cause that only causes appendicitis in 0.0005% of cases [9,10].

The presentation might range from low-grade fever and tenderness on examination [11], and complications from ingesting foreign bodies are rare - less than 1% of cases have reported complications [10].

Acute appendicitis can affect people of any age, but it is most common between the ages of 10 and 20 years, with a lifetime risk of 8.6% in males and 6.9% in females [12]. According to pathophysiology, when the weight of the foreign body exceeds the content of the bowel fluid, it will stick in the lower part of the cecum, where it tends to gather; once it enters the appendix lumen, peristalsis is insufficient to expel the foreign body back to the cecum [9]. The appendicular orifice will enlarge and permit the foreign body's entry into the appendicular lumen [13], which will result in obstruction of the appendicular lumen and trigger an inflammatory process [9]. Bird shot, bullets, fishing line, coins, screws, pins, needles, toothpicks, teeth, keys, and contraceptives are all examples of foreign bodies that might induce acute appendicitis, here in the discussed case it was a needle [14,15]. A previous study demonstrates that all appendicular sites, with the exception of the retroperitoneal appendix, are highly difficult to allow foreign particles to enter the lumen [13].

A blunt foreign body induces irritation of the appendix by an obstruction, whereas elongated and pointy objects, like the one in our instance, are more likely to cause perforation and the development of an abscess [16]. However, a foreign body causing perforation in the digestive tract is very rare, with an incidence of less than 1% [17].

To treat foreign body ingestion, begin with imaging and an urgent upper endoscopy to localize and recover the foreign body, if possible. If the endoscopy was unsuccessful in retrieving the foreign body, then a follow-up with a plain abdominal x-ray is required to show the progression of the foreign body inside the GI tract. Radiographic findings that clinicians will be looking for include gas within the appendix, distention or puffiness of the terminal ilium or cecum, or ascending colon [18].

Other imaging modalities, such as ultrasound, may be ordered to confirm foreign body-induced appendicitis. Some studies have shown that ultrasound reduces the number of unnecessary admissions and appendectomies. A complete blood count could be requested to show evidence of leukocytosis, particularly in asymptomatic cases [18].

The goal of treatment is to reduce the likelihood of complications like perforation because perforation rates are high in sharp and elongated objects, necessitating appendectomy. In previous cases and published reports, authors recommended a prophylactic appendectomy in blunt foreign bodies because it may be associated with perforation [18]. In specific clinical settings, a close follow-up to ensure the spontaneous passage of foreign bodies may be considered an alternative management method [19].

Our case is significant for increasing understanding of appendicitis and its uncommon causes, such as foreign materials. Acute appendicitis is the most frequent surgical emergency worldwide and requires early diagnosis and treatment to avoid complications.

## Conclusions

This rare case of acute appendicitis highlights the importance of considering asymptomatic foreign body ingestion as part of the etiology despite the time gap between the two incidents. Therefore, it necessitates considering appropriate imaging modalities and prophylactic measures that vary depending on the nature of the ingested object and the patient's health status.

## References

[1] (2022). Appendix. https://www.britannica.com/science/appendix.

[2] Baird DL, Simillis C, Kontovounisios C, Rasheed S, Tekkis PP (2017). Acute appendicitis. BMJ.

[3] Humes DJ, Simpson J (2006). Acute appendicitis. BMJ.

[4] Sama CB, Aminde LN, Njim TN, Angwafo FF 3rd (2016). Foreign body in the appendix presenting as acute appendicitis: a case report. J Med Case Rep.

[5] Antonacci N, Labombarda M, Ricci C, Buscemi S, Casadei R, Minni F (2013). A bizarre foreign body in the appendix: a case report. World J Gastrointest Surg.

[6] Packard E, Groff A, Shahid Z, Sahu N, Jain R (2019). A 'bit' of appendicitis: a case of a foreign object in the adult appendix. Cureus.

[7] Simpson J, Humes DJ (2012). Acute appendicitis. Textbook of Clinical Gastroenterology and Hepatology.

[8] Appendicitis. (2023, January 1 (2022). Appendicitis. https://en.m.wikipedia.org/wiki/Appendicitis?fbclid=IwAR1dskAfPOQoM12eA0WS0XlAn0jKVwaen-bmZmwPFSfRzC4jVLw5KnFYJUE.

[9] Klingler PJ, Seelig MH, DeVault KR, Wetscher GJ, Floch NR, Branton SA, Hinder RA (1998). Ingested foreign bodies within the appendix: a 100-year review of the literature. Dig Dis.

[10] Larsen AR, Blanton RH (2000). Appendicitis due to bird shot ingestion: a case study. Am Surg.

[11] Cheng He R, Nobel T, Greenstein AJ (2021). A case report of foreign body appendicitis caused by tongue piercing ingestion. Int J Surg Case Rep.

[12] Baek SK, Bae OS, Hwang I (2012). Perforated appendicitis caused by foreign body ingestion. Surg Laparosc Endosc Percutan Tech.

[13] Wakeley C, Gladstone R (1928). The relative frequency of the various positions of the vermiform appendix as ascertained by an analysis of 5000 cases. Lancet.

[14] Benizri EI, Cohen C, Bereder JM, Rahili A, Benchimol D (2012). Swallowing a safety pin: report of a case. World J Gastrointest Surg.

[15] Hazer B, Dandin O, Karakaş DO (2013). A rare cause of acute appendicitis: an ingested foreign body. Ulus Travma Acil Cerrahi Derg.

[16] Grassi V, Desiderio J, Cacurri A (2016). A rare case of perforation of the subhepatic appendix by a toothpick in a patient with intestinal malrotation: laparoscopic approach. G Chir.

[17] Beh JC, Uppaluri AS, Koh BF, Cheow PC (2016). Fishbone perforated appendicitis. J Radiol Case Rep.

[18] Tustumi F, Hudari GG, Modolo NR, Morrell AL, de Miranda Neto AA, Dias AR (2020). Unusual cause of appendicitis. A case report of acute appendicitis caused by needle ingestion. Int J Surg Case Rep.

[19] Fuller MY, Leino DG, Reyes-Múgica M (2022). Ingested foreign bodies can cause appendicitis and perforation: a multi-institutional case series. Pediatr Dev Pathol.

